# The immune checkpoint receptor LAG3: Structure, function, and target for cancer immunotherapy

**DOI:** 10.1016/j.jbc.2024.107241

**Published:** 2024-03-30

**Authors:** Roy A. Mariuzza, Salman Shahid, Sharanbasappa S. Karade

**Affiliations:** 1 W.M. Keck Laboratory for Structural Biology, University of Maryland Institute for Bioscience and Biotechnology Research, Rockville, Maryland, USA; 2 Department of Cell Biology and Molecular Genetics, University of Maryland, College Park, Maryland, USA

**Keywords:** LAG3, immune checkpoint, MHC, FGL1, structure

## Abstract

Lymphocyte activation gene 3 protein (LAG3) is an immune checkpoint receptor that is highly upregulated on exhausted T cells in the tumor microenvironment. LAG3 transmits inhibitory signals to T cells upon binding to MHC class II and other ligands, rendering T cells dysfunctional. Consequently, LAG3 is a major target for cancer immunotherapy with many anti-LAG3 monoclonal antibodies (mAbs) that block LAG3 inhibitory activity in clinical trials. In this review, we examine the molecular basis for LAG3 function in light of recently determined crystal and cryoEM structures of this inhibitory receptor. We review what is known about LAG3 interactions with MHC class II, its canonical ligand, and the newly discovered ligands FGL1 and the T cell receptor (TCR)–CD3 complex, including current controversies over the relative importance of these ligands. We then address the development and mechanisms of action of anti-LAG3 mAbs in clinical trials for cancer immunotherapy. We discuss new strategies to therapeutically target LAG3 using mAbs that not only block the LAG3–MHC class II interaction, but also LAG3 interactions with FGL1 or TCR–CD3, or that disrupt LAG3 dimerization. Finally, we assess the possibility of developing mAbs that enhance, rather than block, LAG3 inhibitory activity as treatments for autoimmune diseases.

T cells play a major role in generating adaptive immune responses to microbes and cancers. This process is mediated by the T cell receptor (TCR)–CD3 complex, which is composed of a genetically diverse TCR in association with invariant CD3 subunits (1). The TCR mediates recognition of antigenic peptides bound to major histocompatibility complex molecules (pMHC), while the CD3 molecules transmit activation signals to the T cell. The TCR–CD3 complex has evolved to display exquisite sensitivity to pMHC, enabling the host to respond to subtle differences between self and foreign antigens. When activated by pMHC, T cells are extremely effective at orchestrating inflammatory responses and directly destroying host cells that have been infected by a pathogen. While these activities are critical to combat invading microorganisms, there is a risk that bystander host tissue not affected by the pathogen may be injured as well. Moreover, it is also necessary to have ways to thwart T cell responses that have been triggered inappropriately. Accordingly, the immune system has developed several mechanisms to hold T cell responses in check and to downregulate stimulated T cells once a pathogenic challenge has been overcome. Among these mechanisms are immune inhibitory receptors (also called immune checkpoint receptors) expressed on the surface of activated CD4^+^ and CD8^+^ T cells that are upregulated in settings of chronic antigen stimulation or inflammation and act to limit tissue damage and autoimmune-mediated host tissue pathology (2, 3). These T cell immune checkpoint receptors include cytotoxic T-lymphocyte-associated protein 4 (CTLA4), programmed cell death protein 1 (PD1), T cell immunoglobulin and mucin domain-containing protein 3 (TIM3), T cell immunoreceptor with Ig and ITIM domains (TIGIT), and lymphocyte activation gene-3 protein (LAG3; CD223), which is the topic of this review.

In addition to their role in anti-microbial immunity, T cells are the principal mediators of adaptive immune responses to tumors. However, elevated expression of immune checkpoint receptors on exhausted T cells in the tumor microenvironment limits their anti-tumor activity (4, 5). Exhausted T cells are unresponsive to pMHC. Based on this discovery, T cell immune checkpoint receptors are currently major therapeutic targets in cancer, with monoclonal antibodies (mAbs) that block CTLA4 and PD1/PDL1 in the clinic since 2010 (5, 6). Although these mAbs have dramatically improved outcomes for some patients (10–30%), notably for those with metastatic melanoma, the majority of patients do not exhibit long-term durable responses. Therefore, there is an urgent need to identify additional targets to combine with anti-CTLA4 and anti-PD1/PDL1 mAbs to improve their efficacy (7, 8). This has generated intense interest in LAG3, which is the third immune checkpoint receptor to be targeted in the clinic (9). LAG3 downregulates T cell activation, proliferation, and cytokine production, rendering T cells dysfunctional (10).

LAG3 is a ∼55 kDa type I transmembrane glycoprotein comprising four extracellular immunoglobulin (Ig)-like domains (D1–D4) (11, 12), a connecting peptide, and an intracellular region that transmits inhibitory signals to the T cell upon binding to MHC class II and other ligands (7, 13). LAG3 resembles the T cell co-receptor CD4 in that it has a similar domain architecture, is ∼25% identical at the amino acid level, and binds to MHC class II. In addition to MHC class II, its canonical ligand, other proposed LAG3-binding partners include galnectin-3 (Gal-3) (14), liver and lymph node sinusoidal endothelial cell C-type lectin (LSECtin) (15), α-synuclein (16), fibrinogen-like protein 1 (FGL1) (17), and the TCR–CD3 complex (18).

The importance of LAG3 as an immune checkpoint on both effector T cells and regulatory T cells has been demonstrated in multiple disease models, including type 1 diabetes (19), Parkinson’s disease (16), allogeneic bone marrow transplant (20), and cancer (21). In particular, preclinical studies using mAbs to block LAG3 and PD1 inhibitory activity showed significant increases in tumor clearance and survival in several mouse tumor models (22, 23). As a result, nearly 20 anti-LAG3 mAbs are currently in clinical trials for immunotherapy of multiple solid tumors and lymphomas (7, 8, 24). Importantly, a phase II/III randomized trial of one of these mAbs (relatlimab) in combination with the anti-PD1 mAb nivolumab achieved 48% 12-month progression-free survival in advanced melanoma patients, compared with 36% with nivolumab alone (9). Based on these results, the FDA recently made a milestone decision to approve the relatlimab/nivolumab combination for the treatment of unresectable or metastatic melanoma.

An excellent review of LAG3 was recently published that focused on the immunobiology of this receptor, in particular LAG3 signaling, cell-specific functions, and role in different disease settings (25). In this review, we focus on structural and mechanistic aspects of LAG3 function. We first highlight key features of recently determined X-ray crystallographic and cryoEM structures of LAG3. We next review LAG3 ligands and what is known about their interactions with LAG3 and their relative importance in LAG3 function. We then direct our attention to the development and mechanisms of action of antagonist mAbs targeting LAG3 for cancer immunotherapy, including new therapeutic approaches. Finally, we explore the possibility of developing LAG3 agonists to treat autoimmune and inflammatory diseases (26).

### Structure of LAG3

Crystal structures have been reported for the full-length ectodomain (D1–D4) of human LAG3 (hLAG3) bound to the Fv (an antibody fragment consisting of V_L_ and V_H_ domains) of an antagonist mAb (F7) (12), D1–D4 of mouse LAG3 (mLAG3) (27), D1–D2 of mLAG3 (12), and D3–D4 of hLAG3 bound to Fv F7 (12). In addition, the cryoEM structure of hLAG3 (D1–D4) in complex with the Fab of a therapeutic mAb (favezelimab) has been determined (28). However, no structural information is available for LAG3 bound to MHC class II, FGL1, or other biological ligand.

Both hLAG3 and mLAG3 crystallized as parallel homodimers mediated by mainly hydrophobic contacts across a relatively small D2–D2 interface comprising ∼450 Å^2^ of buried surface area (Fig. 1*A*) (12, 27). In agreement with the crystal structures, dimerization of hLAG3 and mLAG3 in solution was demonstrated by size exclusion chromatography coupled with multi-angle light scattering (SEC-MALS) (12). However, mLAG3 dimers appeared more stable than hLAG3 dimers, based on the distinct elution profiles of the two proteins. By contrast, CD4 is monomeric both in crystal structures and in solution (29). Moreover, the ability of mLAG3 to dimerize on the cell surface was established by flow cytometric Förster resonance energy (flow-FRET) assays of HEK293 cells transfected with fluorescent LAG3 constructs (27), consistent with an earlier study (30).

**Figure 1:**
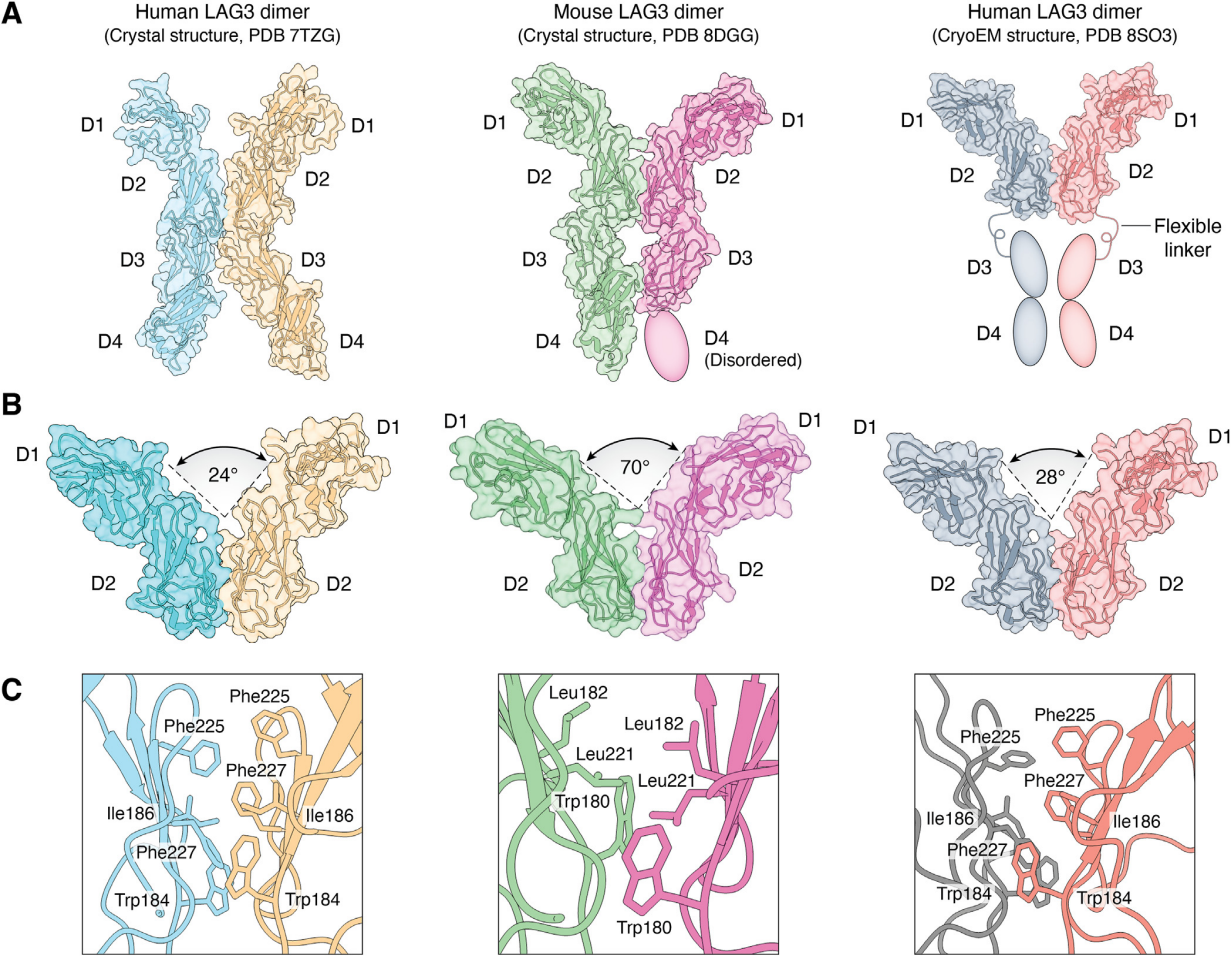
**Structure of LAG3.***A*, overall structure of human and mouse LAG3 dimers. *Left*, LAG3 monomers in the crystal structure of human LAG3 are *cyan* and *yellow* (PDB accession code 7TZG) (12). Domains D1–D4 are labeled. *Middle*, LAG3 monomers in the crystal structure of mouse LAG3 are *green* and *magenta* (8DGG) (27). D4 of one monomer is disordered. *Right*, LAG3 monomers in the cryoEM structure of human LAG3 are *gray* and *salmon* (8SO3) (28). D3 and D4 of both monomers are not visible due to flexibility in the linker in the linker connecting D2 and D3. *B*, dimerization of LAG3 is mediated by D2–D2 interactions in both crystal and cryoEM structures. Dihedral angles between D2 β-strands are indicated. Mouse LAG3 has a wider V-shaped architecture than human LAG3. *C*, close-up views of D2–D2 interfaces. Contacting residues are labeled.

The four Ig-like domains of both hLAG3 and mLAG3 adopt an elongated arrangement resembling that of CD4 (Fig. 2) (12, 27). D1 is V-type domain while D2, D3, and D4 are C2-type domains. Whereas D1 and D2 fold as discrete domains, D3 and D4 form a contiguous unit linked by an extended β-strand. In the cryoEM structure of hLAG3, no electron density was visible for D3 or D4, which likely reflects flexibility in the linker connecting D2 and D3 (Fig. 1*A*) (28). By contrast, the ordered nature of all four Ig-like domains in crystal structures of LAG3 D1–D4 (12, 27) is probably due to capture of a particular LAG3 conformation by crystal lattice contacts. In both crystal and cryoEM structures, a proline-rich 25-residue loop that connects the C and C' β-strands of the D1 domain (designated the D1 loop; residues 74–98 in hLAG3) is mostly disordered, implying flexibility. The D1 loop is not found in CD4 and constitutes at least part of the binding site for MHC class II (31) and some therapeutic mAbs (32), as discussed below.

**Figure 2:**
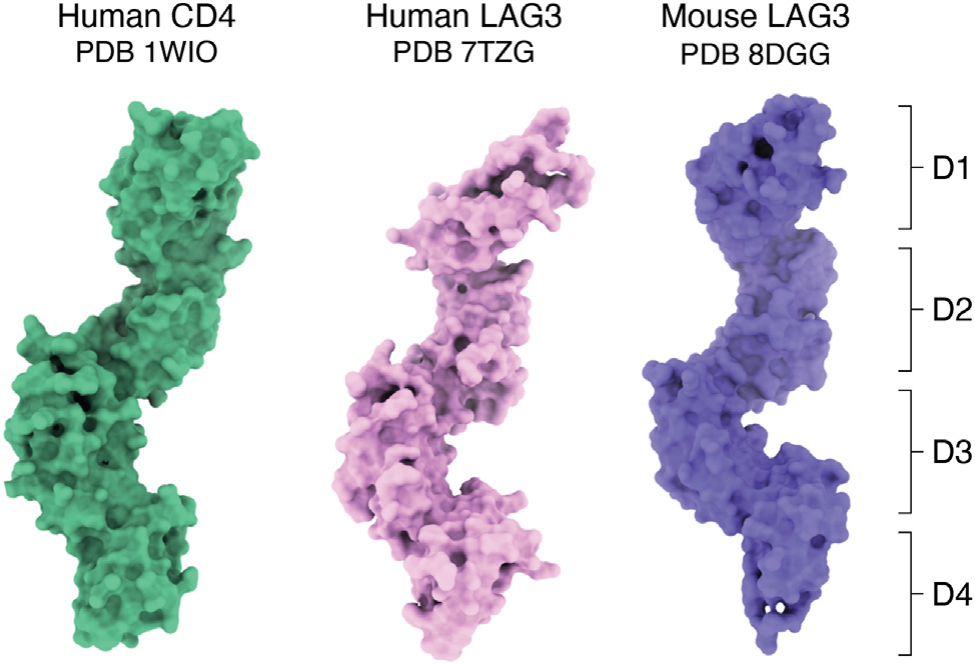
**Comparison of LAG3 and CD4 structures.** D1–D4 of LAG3 (7TZG and 8DGG) and CD4 (1WIO) adopt similar elongated conformations (surface representations).

LAG3 forms a V-shaped dimer *via* D2–D2 interactions in both crystal and cryoEM structures (Fig. 1*B*) (12, 27, 28). Dimerization of hLAG3 is mediated by a cluster of hydrophobic residues (Trp184, Ile186, Phe225, and Phe227) that are analogous to Trp180, Ile182, and Leu221 of mLAG3 (Fig. 1*C*) (12). Although hLAG3 uses the same set of residues for dimerization in the crystal and cryoEM structures, they make different interactions across the D2–D2 interface in the two dimers, possibly due to favezelimab binding in the cryoEM structure (28). The hLAG3 dimer is considerably narrower than the mLAG3 dimer due to a twist angle of 24° instead of 70° (Fig. 1*B*). However, it is unknown whether these different conformations represent species-specific structural features or distinct functional states.

#### LAG3 dimerization is required for inhibitory activity

A recent study has revealed that LAG3 dimerization is critical for LAG3 function (27). Wild-type (dimeric) LAG3 associates with TCR–CD3 complexes and co-localizes to the immunological synapse (IS) following T cell activation (18), as discussed later. The IS is the interface between a T cell and an antigen-presenting cell. To disrupt the dimerization of mLAG3, Trp180 and Leu221, which occupy opposite positions in the D2–D2 interface (Fig. 1*C*), were mutated to glutamate (27). Mutated mLAG3 was predominantly monomeric in solution, whereas the wild-type protein was dimeric, as demonstrated by SEC-MALS (12). In agreement with solution studies, flow cytometry-based Förster resonance energy transfer assays showed that wild-type mLAG3, but not mutated mLAG3, dimerized on the surface of transfected Expi293 cells (27). LAG3 dimerization was found to be essential for binding MHC class II and FGL1 ligands, as demonstrated by the inability of T cells expressing monomeric, but not dimeric, LAG3 to bind MHC class II tetramers or FGL1 dimers in flow cytometric assays (27). Moreover, loss of binding resulted in loss of LAG3 inhibitory function in the context of T cell activation by cognate peptide–MHC, highlighting the critical role of LAG3 dimerization in suppressing T cell responses.

### LAG3 ligands

Multiple ligands may engage the ectodomain of LAG3 to stimulate its inhibitory function (Fig. 3). These include MHC class II (33), Gal-3 (14), LSECtin (15), α-synuclein (16), FGL1 (17), and the TCR–CD3 complex (18), as discussed below. However, their hierarchy in terms of functional importance is unknown. Additionally, it remains controversial whether MHC class II alone is responsible for the immunosuppressive activity of LAG3.

**Figure 3:**
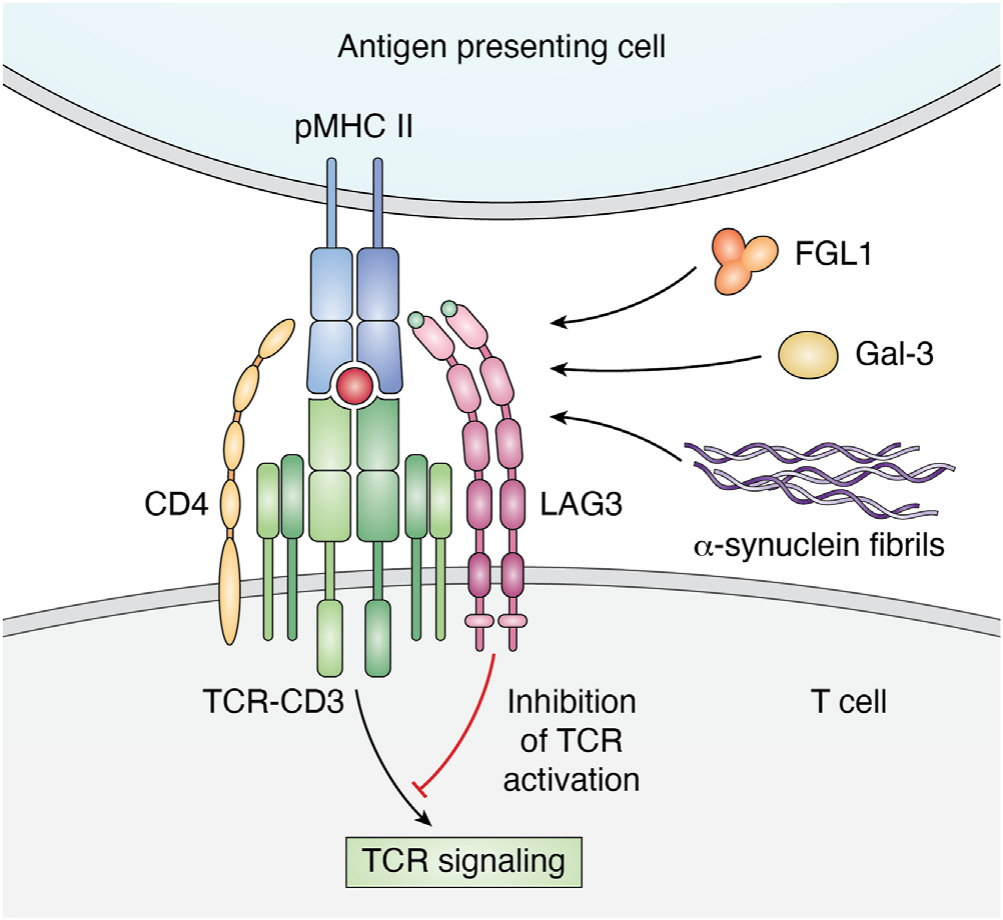
**LAG3 ligands.** The role of LAG3 under normal conditions is to limit TCR signaling and downregulate stimulated T cells once a pathogenic challenge has been overcome. LAG3 is dimeric and CD4 is monomeric. TCR ligands include MHC class II, Gal-3, α-synuclein, FGL1, and the TCR–CD3 complex. The *red ball* represents the antigenic peptide presented by MHC class II to TCR. The *green ball* on the N-terminal D1 domain of LAG3 represents a unique 25-residue loop not found in CD4. The LAG3 D1 loop contains at least part of the binding site for MHC class II and is the target of some therapeutic mAbs.

#### Interaction of LAG3 with MHC class II

LAG3 was discovered in 1990 in the form of a transcript expressed by an IL-2-dependent natural killer cell line that encoded a transmembrane protein with ∼25% amino acid sequence identity to CD4 (11). The LAG3 gene is adjacent to the CD4 gene (chromosome 12 in humans). The chromosomal location of these genes and their similar exon/intron organization indicated that they share an ancient ancestor. The structural homology of LAG3 to CD4 suggested that LAG3, like CD4, would bind MHC class II (11). This expectation was confirmed using cell-cell adhesion assays (34). However, the affinity of LAG3 for MHC class II is ∼1000-fold greater than that of CD4 (33). As measured by surface plasmon resonance (SPR), hLAG3 bound to HLA-DR4 and HLA-DP2 MHC class II molecules loaded with class II-associated invariant chain peptide (CLIP) with dissociation constants (*K*_D_s) of 2.5 and 3.1 μM, respectively, while mLAG3 bound to CLIP-loaded I-A^b^, I-A^d^, and I-A^g7^ MHC class II molecules with *K*_D_s of 4.9, 4.8, and 1.9 μM, respectively (12). Thus, LAG3, like CD4, has the remarkable ability to recognize highly polymorphic MHC class II molecules with only minor variations in affinity. In the case of CD4, this ability is explained by the targeting *via* its D1 domain of invariant residues at a concave site formed by the α2 and β2 of domains of MHC class II molecules that is beneath the peptide-binding site (Fig. 4*A*) (35). Although a similar solution to MHC cross-recognition might be expected for LAG3, LAG3 and CD4 do not compete for binding to MHC class II, as demonstrated by the simultaneous binding of LAG3 and CD4 D1–D4 constructs to MHC class II on Raji lymphoblast-like cells (13). This surprising result indicates that LAG3 and CD4 bind to distinct, non-overlapping sites on MHC class II and that LAG3 does not inhibit T cell activation by outcompeting CD4 for MHC class II binding, but probably through a mechanism whereby LAG3 disrupts CD4–Lck interactions at the IS (18) (see below). LAG3 was shown by mutagenesis and cell–cell adhesion assays to engage MHC class II through its D1 domain, as in the case of CD4 (35), with most binding residues clustered at the base of the D1 loop (31). However, a full description of LAG3–pMHC class II interactions must await structure determination of the corresponding complex.

**Figure 4:**
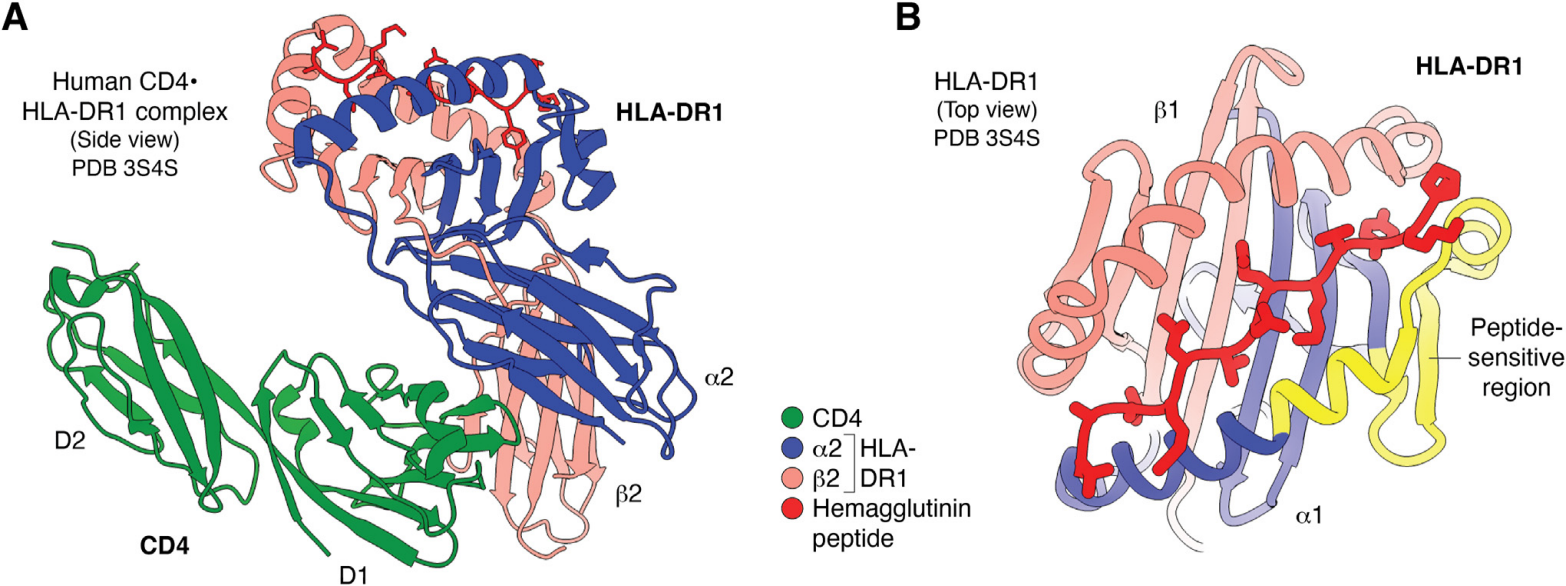
**Interaction of CD4 and LAG3 with MHC class II.***A*, structure of the complex between human CD4 and HLA-DR1 (*side view*) (3S4S) (36). CD4 (*green*) contacts both the α2 (*blue*) and β2 (*salmon*) domains of the MHC class II molecule through its D1 domain. An influenza hemagglutinin peptide bound to HLA-DR1 is *red*. *B*, structure of HLA-DR1 (*top view*) showing the region (*yellow*) influenced by the stability of the peptide–MHC class II complex. This peptide-sensitive region, which is distant from the CD4 binding site but could represent the LAG3 binding site, comprises residues with a root-mean-square deviation of >2 Å *versus* HLA-DR1 bound to the peptide exchange catalyst HLA-DM (38, 39).

Another surprising feature of LAG3 that is not shared with CD4 is its selective recognition of conformationally stable pMHC class II complexes (13). Moreover, LAG3 requires such complexes for inhibition of T cell responses. Using a pentameric form of mLAG3 as a staining reagent in flow cytometry, Maruhashi *et al.* (13) found that some cell lines expressing large amounts of MHC class II nevertheless bound little or no LAG3. They then carried out expression cloning to identify the interferon-γ-inducible transcriptional coactivator CIITA as the factor necessary for LAG3 binding. CIITA induces the expression of proteins such as Ii and H2-DM that are involved in the assembly and cell surface expression of stable pMHC class II complexes. Furthermore, MHC class II expressed with covalently attached high-affinity peptides bound strongly to LAG3 in a CIITA-independent manner, whereas MHC class II linked to low-affinity peptides did not (13). At the functional level, high-affinity peptides elicited much more pronounced LAG3-mediated inhibitory effects on T cells than low-affinity peptides. On the other hand, LAG3 did not distinguish between CLIP–HLA-DR4 and CLIP–HLA-DP2 complexes (12) probably because these pMHC complexes are similarly stable.

The biological role of LAG3’s unexpected peptide selectivity remains to be elucidated. One possibility is that LAG3 may suppress autoimmunity by inhibiting autoreactive T cells recognizing stable autoantigen–MHC class II in the periphery that somehow escaped thymic negative selection (13). In support of this hypothesis, LAG3 was shown to selectively inhibit the activation of diabetogenic CD4^+^ T cells recognizing stable autoantigen–MHC class II in non-obese diabetic (NOD) mice, resulting in slower onset and lower penetrance of type 1 diabetes (T1D) than in NOD mice deficient in LAG3 (19, 36, 37).

Although we do not understand the structural basis for the preferential binding of LAG3 to more stable pMHC class II complexes, it could involve peptide-dependent variations in MHC class II conformation (38). For example, a comparison of stable peptide–HLA-DR complexes with HLA-DM-stabilized HLA-DR in a complex with a low-affinity peptide revealed that stable peptide binding induces conformational changes in the floor of the peptide-binding groove and at one end of the α1 helix that flanks the groove (Fig. 4*B*) (39). LAG3 could conceivably sense pMHC complex stability by binding to this site, which is distinct from the CD4 binding site (Fig. 4*A*). Consistent with this idea, LAG3 and CD4 bind non-overlapping sites on MHC class II (13), as noted above. Another possibility is that LAG3 senses pMHC complex stability through a process known as dynamic allostery (40, 41, 42) whereby peptide binding alters MHC flexibility without obvious structural changes in the protein. Indeed, different peptides have been found to alter the energetic landscape of MHC class I, impacting motions throughout the protein (43, 44). Dynamic allostery is believed to explain the peptide sensitivity of the NK receptor Ly49C, which binds MHC class I at a site distant from the peptide-binding site with no evidence of binding-induced conformational changes in crystal structures of Ly49C–pMHC class I complexes (45). Clearly, the structure of a LAG3–pMHC class II complex is required to establish the molecular basis for LAG3 peptide selectivity.

#### Interaction of LAG3 with FGL1

FGL1 belongs to the fibrinogen family of proteins but lacks the characteristic platelet-binding and thrombin-sensitive sites required for clot formation. It is a disulfide-linked dimer that forms oligomers in solution (17, 46). FGL1 consists of an N-terminal coiled-coil domain that mediates oligomerization and a C-terminal fibrinogen-like domain (FD). Under normal conditions, FGL1 expression is mainly limited to the liver where it is secreted by hepatocytes (47). FGL1 contributes to the metabolic functions of the liver (48, 49, 50, 51), and possibly to its immune-privileged state (17). FGL1 expression is upregulated in human solid tumors, including melanoma, lung cancer, prostate cancer, and colorectal cancer, with the highest percentage upregulation (35%) in lung cancer (17). High plasma FGL1 levels are associated with poor outcomes in cancer patients undergoing anti-PD1 therapy, suggesting that it might contribute to tumor resistance.

Wang *et al.* (17) recently identified FGL1 as a ligand for LAG3 by high-throughput screening of nearly 6000 transmembrane proteins expressed on transfected HEK293 cells using a soluble LAG3 D1–D4 construct. In that study, FGL1 was found to bind mLAG3 with high affinity (*K*_D_ ∼ 1.5 nM), as measured by biolayer interferometry (BLI) using immobilized LAG3 ectodomain and soluble FGL1 as the analyte. However, another study reported a much lower affinity (*K*_D_ ∼ 7 μM) (52). This large difference (∼5000-fold) is probably due to differences in the oligomeric state of the particular FGL1 preparations used for affinity measurements. FGL1 is known to form multimers in solution (17, 46), which could produce large avidity effects in BLI experiments.

Wang *et al.* (17) showed that recombinant FGL1 inhibited antigen-specific T cell activation *in vitro*. Moreover, administration of a mAb against FGL1 enhanced antigen-specific T cell activation in TCR OT-1 transgenic mice immunized with ovalbumin peptide antigen in a manner similar to the anti-LAG3 mAb C9B7W. Mice deficient in FGL1 slowly developed spontaneous autoimmune symptoms (17), consistent with a role for FGL1 as an immune suppressive molecule. In addition, silencing the LAG3–FGL1 interaction by either genetic knockout or antibody blockade promoted anti-tumor immunity, as manifested by reduced growth of MC38 colon carcinoma cells in inoculated mice.

Despite these findings, the functional relevance of FGL1 interactions with LAG3 has been challenged. In contrast to results from Wang *et al.* (17), Maruhashi *et al.* (52) found that FGL1 binding to LAG3 failed to induce or augment the inhibitory effect of LAG3 on the activation of CD4^+^ or CD8^+^ T cells. Moreover, LAG3 mutants lacking FGL1-binding capacity but not mutants unable to bind MHC class II potently suppressed T cell activation, indicating that FGL1 binding is dispensable for LAG3 to inhibit T cell activation, at least *in vitro*. The relative importance of FGL1 and MHC class II as functional ligands for LAG3 was assessed *in vivo* in NOD mice (52), in which LAG3 strongly suppresses diabetogenic T cells to avoid fulminant T1D (36, 53). Mice harboring the LAG3 P111A mutation, which abolishes MHC class II binding without affecting FGL1 binding, developed fulminant T1D similar to LAG3-deficient mice (52). However, mice harboring the LAG3 K27E mutation, which abolishes FGL1 binding without affecting MHC binding, did not experience exacerbation of T1D, implying that FGL1 is not required for LAG3-mediated mitigation of autoimmunity, at least in the NOD model.

The reasons for the apparent discrepancies between the studies of Wang *et al.* (17) and Maruhashi *et al.* (52) are unclear. It is possible, for example, that the LAG3 K27E mutation tested in NOD mice did not completely abrogate LAG3–FGL1 interactions under physiological conditions *in vivo* and that FGL1 may still contribute to limiting autoimmunity. Further studies are obviously called for to investigate the functional relevance of LAG3–FGL1 interactions.

The FGL1 binding site of LAG3 was mapped by identifying mutations that diminished or increased the affinity of hLAG3 for FGL1 (12). These mutations revealed an extended binding surface, mainly on D1, that includes a flexible loop (designated loop 2) between the C’ and D β-strands of D1 (Fig. 5*A*). The analogous region of CD4 forms the C" strand of D1 that contains multiple MHC class II-contacting residues (35). Additionally, the LAG3 P111A mutation that abolishes MHC class II binding (13) is located in loop 2, indicating a functional role for this site in both LAG3 and CD4. However, the P111A mutation did not affect binding of LAG3 to FGL1, indicating that MHC class II and FGL1 bind to distinct, though possibly overlapping, regions of LAG3 (52).

**Figure 5:**
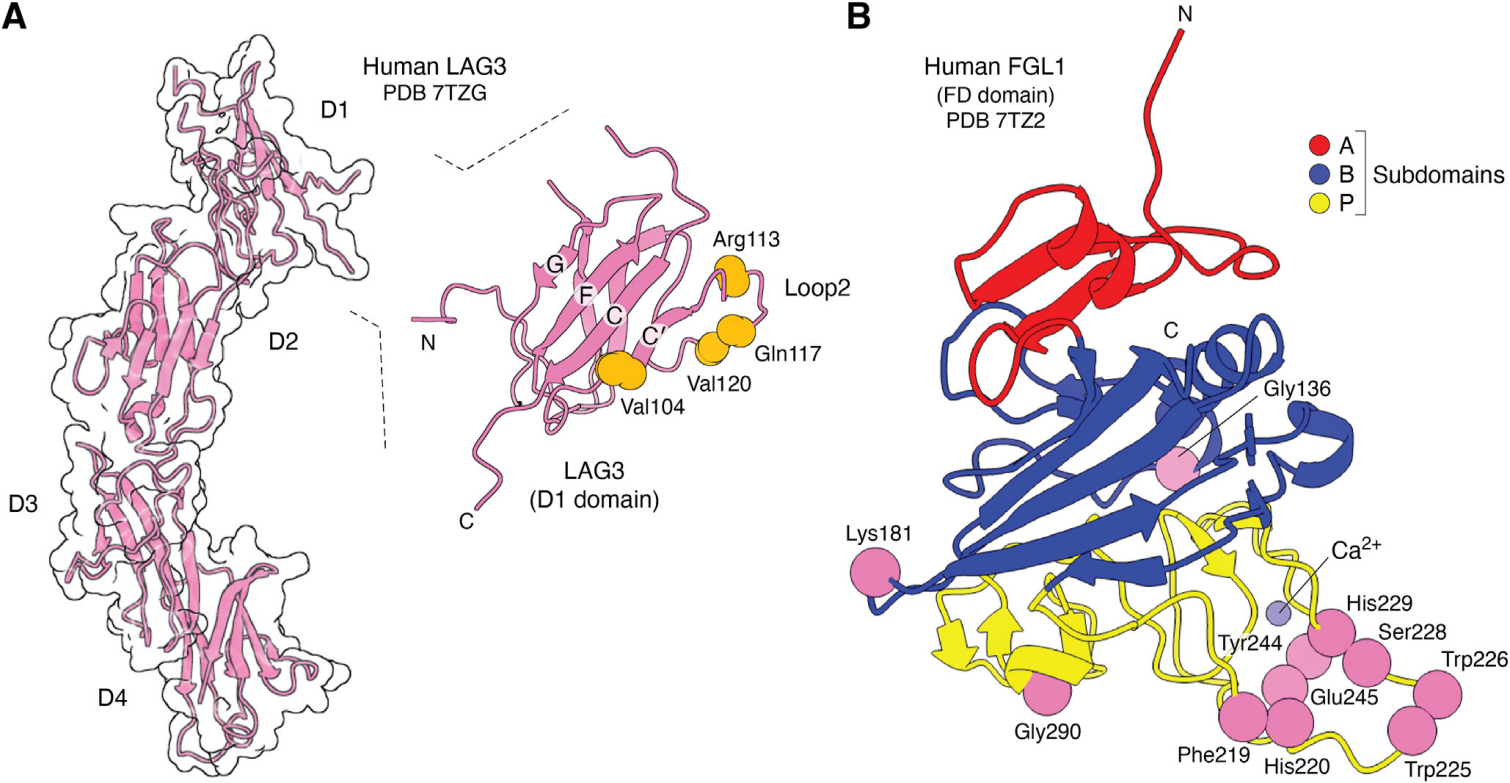
**Interaction of LAG3 with FGL1.***A*, residues in the D1 domain of human LAG3 implicated in binding FGL1 (12). *B*, crystal structure of the C-terminal fibrinogen-like domain of human FGL1 (7TZ2) (12). The A, B, and P subdomains are *red*, *blue*, and *yellow*, respectively. Residues in the P subdomain that positively or negatively affect FGL1 binding to LAG3 are indicated.

The crystal structure of the FD of FGL1 revealed three well-defined subdomains (A, B, and P) that pack sequentially in the globular body of the structure, with a calcium-binding site located in the P subdomain (Fig. 5*B*) (12). The FGL1 FD is most similar to the FDs of angiopoietin-1 and fibrinogen C domain-containing protein 1. The LAG3 binding site of FGL1 was mapped by identifying mutations that reduced or enhanced the affinity of FGL1 for LAG3 (12). These mutations cluster in the P subdomain (Fig. 5*B*). Notably, this putative LAG3 binding surface includes several loops that mediate ligand recognition by other P subdomains (54, 55). Ming *et al.* (12) showed that FGL1 binding cross-links LAG3 on the surface of Jurkat cells to induce LAG3 clustering. LAG3 clustering may serve as a mechanism for LAG3-mediated T cell suppression by soluble FGL1 ligand.

#### TCR–CD3 as a *cis*-acting LAG3 ligand

Typically, cell surface receptors bind ligands expressed on other cells to mediate cell–cell communication (in *trans*), as exemplified by the interaction of LAG3 with MHC class II. However, an increasing number of cell surface receptors are known to also interact with ligands expressed on the same cell (in *cis*) (56, 57). Examples include CD22 (Siglec-2), a negative regulator of B cell signaling that recognizes sialic acid modifications of surface glycoproteins in *cis* and *trans* (58, 59), and the NK cell receptors Ly49 and LILR, which can interact with MHC class I ligands expressed on the same or opposing cells (60, 61).

LAG3 was recently shown to constitutively associate in *cis* with the TCR–CD3 complex on the surface of CD4^+^ and CD8^+^ T cells in an MHC class II-independent manner (Fig. 3) (18). Following TCR stimulation, LAG3 migrated with TCR–CD3 to the IS and limited TCR signaling and T cell proliferation in the absence of MHC class II ligation. These findings suggest that TCR–CD3 serves as a *cis* ligand for LAG3 (18). However, they do not preclude a role for MHC class II, which may be critical to amplifying LAG3 function when LAG3 concentrations on T cells are limiting. The regions of LAG3 that interact with TCR–CD3, and of TCR–CD3 that interact with LAG3, remain to be determined.

Mechanistically, LAG3 was shown to exert its inhibitory activity on T cells by disrupting the interaction between the co-receptors CD4 and CD8 and the tyrosine kinase Lck, thereby reducing phosphorylation of ZAP70 and limiting signaling downstream of the TCR–CD3 complex following stimulation (Fig. 3) (18). CD4 and CD8 bind non-covalently to Lck *via* a unique dicystine motif that coordinates Zn^2+^ (62). The cytoplasmic tail of LAG3 contains a phylogenetically conserved, highly repetitive glutamic acid–proline tandem repeat named the EP motif. Guy *et al.* (18) discovered that the negatively charged LAG3 EP motif binds Zn^2+^ and disrupts Zn^2+^-dependent interactions between the CD4 and CD8 co-receptors and Lck. The EP motif sequesters Zn^2+^ by mediating a reduction of local pH within the IS, where LAG3 co-localizes with the TCR–CD3 complex after T cell activation. This in turn restricts ZAP70 phosphorylation and inhibits downstream TCR signaling.

#### Other potential LAG3 ligands

In addition to MHC class II, FGL1, and TCR–CD3, three other LAG3 ligands have been proposed: Gal-3 (14), LSECtin (15), and α-synuclein (16). However, only limited data support the biological relevance of these ligands. Gal-3 is a galactose-binding lectin secreted by tumor cells whose interaction with LAG3 was reported to suppress CD8^+^ T cell function (14). LSECtin expressed on melanoma cells was found to inhibit interferon-γ production by antigen-specific effector T cells, possibly by engaging carbohydrates on LAG3 (15). However, lectin receptors are notoriously difficult to validate and further work is required to establish Gal-3 and LSECtin as *bona fide* LAG3 ligands.

LAG3 has been implicated in the pathogenesis of Parkinson’s disease by mediating neuron-to-neuron transmission of misfolded preformed fibrils (PFF) of α-synuclein (16). LAG3 bound α-synuclein PFF with high affinity (*K*_D_ ∼ 80 nM), whereas α-synuclein monomer exhibited minimal binding. This interaction was reported to initiate α-synuclein PFF endocytosis, transmission, and neuronal cell toxicity (16). However, this study did not rule out possible contributions by other α-synuclein PFF binding partners, for example, neurexin 1β (63). In addition, a more recent study found no evidence of LAG3 expression in neurons or of a role for LAG3 in modulating α-synucleinopathies (64). Resolving these seemingly contradictory results will require additional investigation.

### LAG3 as a target for cancer immunotherapy

Immune checkpoint inhibitors have become a central pillar in the treatment of patients with cancer (5, 6). Unfortunately, the benefit of mAbs targeting PD1 (pembrolizumab, nivolumab, and cemiplimab), PDL1 (atezolizumab, avelumab, and durvalumab), or CTLA4 (ipilimumab and tremelimumab) is still limited to select patients and most of them either gain minimal benefit or eventually relapse, thereby creating an urgent need for new therapeutic agents to combine with anti-PD1, anti-PDL1, or anti-CTLA4 mAbs (7, 8). This has directed considerable attention to LAG3, the third immune checkpoint receptor to be exploited for cancer immunotherapy. Indeed, nearly 20 anti-LAG3 mAbs or LAG3 bispecifics are now in over 100 clinical trials for treating multiple solid tumors and lymphomas (Table 1) (7, 8, 24). These trials evaluate anti-LAG3 mAbs alone or combined with anti-PD1 mAbs, or as anti-LAG3/anti-PD1, anti-LAG3/anti-PD1L, or anti-LAG3/anti-CTLA4 bispecifics. In 2022, one of these anti-LAG3 mAbs, relatlimab, received FDA approval for treating unresectable or metastatic melanoma in combination with the anti-PD1 mAb nivolumab (https://www.fda.gov/drugs/resources-information-approved-drugs/fda-approves-opdualag-unresectable-or-metastatic-melanoma) (9). In a phase I clinical trial of the anti-LAG3/anti-PD1 bispecific tebotelimab, objective responses were observed in multiple solid tumor types, including PD1-refractory disease, and in LAG3^+^ non-Hodgkin lymphomas (65).

**Table 1:** Anti-LAG3 antibodies in clinical trials

Antibody	Company	Description	Status	Tumor types	Epitope	References
Relatlimab (BMS-986016)	Bristol-Myers Squibb	anti-LAG3	Approved	metastatic melanoma	D1	NCT03743766 NCT05498480 (80)
Favezelimab (MK-420/22D2)	Merck	anti-LAG3	Phase III	cutaneous squamous cell carcinoma, endometrial cancer, metastatic colorectal cancer	D1	NCT06036836 NCT05064059
Fianlimab (REGN3767)	Regeneron	anti-LAG3	Phase II/III	advanced non-small cell lung cancer	ND	NCT05785767
Sym022	Symphogen	anti-LAG3	Phase I	metastatic cancer, solid tumors, lymphomas		NCT03489369
Tuparstobart (INCAGN02385)	Incyte	anti-LAG3	Phase I/II	solid tumors, lung, endometrial, breast, gastric, melanoma and ovarian cancers	ND	NCT05287113
GSK2831781 (IMP731)	GlaxoSmithKline	anti-LAG3	Phase II	psoriasis, ulcerative colitis		NCT02195349 NCT03893565
TSR-033	Tesaro (GSK)	anti-LAG3	Phase I	advanced solid tumors		NCT03250832 NCT02817633 NCT03250832
Ieramilimab (LAG525/IMP701/BAP050)	Immutep/Novartis	anti-LAG3	Phase I/II	neuroendocrine tumors, small-cell lung cancer and diffuse large B cell lymphoma, melanoma, renal cell cancer, mesothelioma	D1	NCT02460224
Miptenalimab (BI-754111/496G6)	Boehringer Ingelheim	anti-LAG3	Phase II	advanced and/or metastatic solid tumors, non-small cell lung cancer	D1	NCT03156114, NCT03433898 (81)
Tebotelimab (MGD013)	MacroGenics	anti-LAG3/anti-PD1bispecific	Phase II/III	ovarian cancer, breast cancer, mesothelioma, gastric cancer, non-Hodgkins lymphomas	ND	NCT03219268 (65)
EMB-02	EpimAb Biotherapeutics	anti-LAG3/anti-PD1bispecific	Phase I/II	advanced solid tumors	ND	NCT04618393
IBI323	Innovent Biologic	anti-LAG3/anti-PD1bispecific	Phase I	advanced malignancies	ND	(82)
CB213	Crescendo Biologics	anti-LAG3/anti-PD1bispecific	Phase I	solid tumors	ND	(83)
Tobemstomig (RG-6139/RO-7247669)	Hoffmann-La Roche	anti-LAG3/anti-PD1bispecific	Phase II	metastatic melanoma, non-small cell lung cancer, esophageal squamous cell carcinoma	ND	NCT04140500
FS118	F-Star Therapeutics	anti-LAG3/anti-PDL1 bispecific	Phase I/II	advanced malignancies	ND	NCT03440437
ABL501	ABL Bio	anti-LAG3/anti-PDL1bispecific	Phase I	advanced solid tumors	ND	NCT05101109 (84)
Pavunalimab (XmAb841)	Xencor	anti-LAG3/anti-CTLA4 bispecific	Phase I	cervical, pancreatic, and hepatocellular carcinomas, triple negative breast cancer	ND	NCT03849469

Abbreviation: ND, not determined.

#### Mechanisms and epitopes of therapeutic antibodies

Most, if not all, therapeutic anti-hLAG3 mAbs in Table 1 were generated in a similar way using standard hybridoma technology to obtain mouse mAbs that were subsequently humanized (66, 67, 68, 69, 70). In a typical approach, mice were immunized with recombinant hLAG3–Fc fusion proteins and hybridoma supernatants screened for binding to hLAG3 expressed on the surface of CHO cells. The anti-hLAG3 mAbs bound to primary human T cells and blocked the binding of hLAG3–Fc to Raji or Daudi B lymphoblast cells expressing MHC class II (66, 67, 68, 69, 70). Epitope mapping of seven anti-hLAG3 mAbs using chimeras between hLAG3 and mLAG3 found that they all target the D1 domain (32).

This focus on D1 could be due to greater immunogenicity of D1 than D2–D4 in mice immunized with hLAG3–Fc. Alternatively, mAbs against D2–D4 may have been discarded during the selection process because they did not inhibit the binding of hLAG3–Fc to cells expressing MHC class II. Indeed, cell–cell adhesion assays using hLAG3 mutants previously localized the MHC class II binding site to the D1 domain (31). It is not known whether any of the anti-hLAG3 mAbs in Table 1 block FGL1 binding, interfere with the interaction of LAG3 with TCR–CD3, or disrupt LAG3 dimerization. Nevertheless, most anti-hLAG3 mAbs now in the clinic, including relatlimab, probably restore T cell effector function by preventing LAG3 from binding MHC class II.

Although most, if not all, therapeutic anti-hLAG3 mAbs target D1, they recognize different epitopes (32). Some almost exclusively target the 25-residue D1 loop that connects the C and C' β-strands of D1, whereas others mainly recognize D1 determinants outside this loop. The precise epitope recognized by one of these mAbs, favezelimab (Table 1) (67, 71) has been delineated by cryoEM (Fig. 6*A*) (28). Favezelimab primarily targets the base of the D1 loop of hLAG3, where it forms a dense hydrogen bonding network with Arg95, Arg97, and Arg98, all *via* V_L_. Additional contacts with the main body of the D1 domain are mediated by V_H_. In this way, favezelimab prevents LAG3 binding to MHC class II (67), which constitutes its mechanism of action. Except for favezelimab, there is no atomic-level structural information linking the epitopes bound by any of the clinical mAbs in Table 1 to their effect on LAG3 activity.

**Figure 6:**
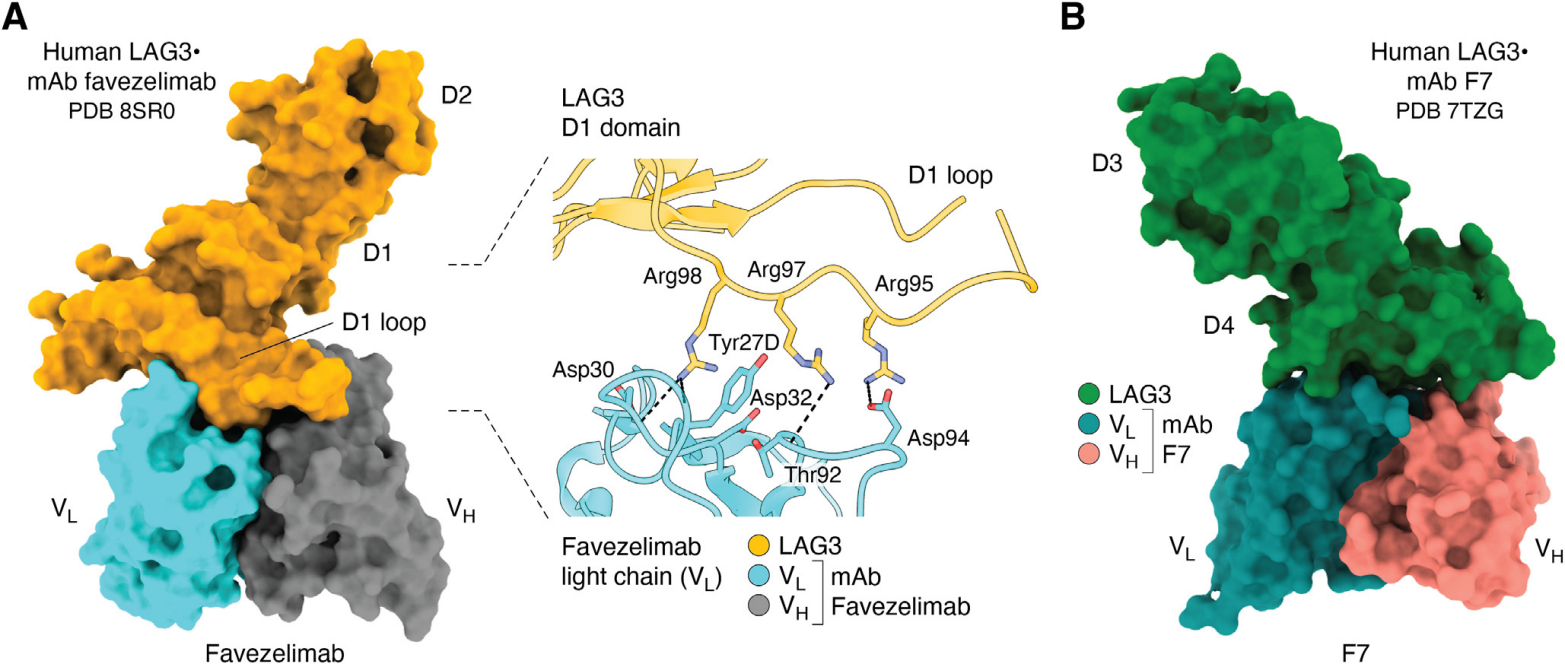
**Structure of LAG3 bound to antagonist antibodies.***A*, CryoEM structure of human LAG3 in complex with the therapeutic mAb favezelimab (8SR0) (28). LAG3 (monomer) is *orange*, V_L_ is *cyan*, and V_H_ is *gray* (surface representation). *Inset* shows interactions between LAG3 and favezelimab. The side chains of contacting residues are drawn in stick representation with carbon atoms in *orange* (LAG3) or *cyan* (V_L_), nitrogen atoms in *blue*, and oxygen atoms in *red*. Hydrogen bonds are indicated by *black dashed lines*. *B*, crystal structure of mAb F7 bound to the D4 domain of human LAG3 (7TZG) (12). LAG3 (monomer) is *green*, V_L_ is *teal*, and V_H_ is *salmon* (surface representation).

#### Development of new therapeutics targeting LAG3

Although the current generation of clinical mAbs were mostly selected for their ability to block binding of LAG3 to its canonical MHC class II ligand, the recent identification of other LAG3 ligands, notably FGL1 and TCR–CD3, opens new possibilities for therapeutically targeting LAG3. In addition, several mAbs have been described that act as LAG3 antagonists without blocking LAG3–MHC class II interactions. One of these, F7, was isolated from a phage library displaying single-chain Fv fragments rather than from a hybridoma (72). As revealed by the crystal structure of Fv F7 in complex with hLAG3 (Fig. 6*B*), F7 engages the juxtamembrane D4 domain of LAG3 (12). While its mechanism of action is unknown, F7 could conceivably interfere with *cis* association of LAG3 with TCR–CD3 complexes (18), thereby preventing T cell suppression without blocking MHC class II binding.

A particularly intriguing mAb is C9B7W, which potently inhibits LAG3 function in multiple *in vitro* and *in vivo* mouse models (18, 23, 27, 36, 73). C9B7W binds to the D2 domain of mLAG3 but has no or limited capacity to block LAG3 interactions with MHC class II (73, 74). Instead, C9B7W was shown to disrupt mLAG3 dimers in solution and on the T cell surface (27). This suggests that C9B7W blocks LAG3 inhibitory activity by disrupting LAG3 dimerization and subsequent binding to MHC class II and FGL1 ligands. Antagonist mAbs directed against domains D1 (M8-4-6) and D3 (410C9) of mLAG3 have also been reported to cause dimer disruption (27).

Given the discovery of FGL1 and TCR–CD3 as LAG3 ligands (17, 18), and the importance of LAG3 dimerization for LAG3 activity (27), the search for clinical LAG3 antagonists should be broadened beyond the present focus on LAG3–MHC class II blockade to include mAbs that disrupt LAG3 interactions with FGL1 and TCR–CD3 as well as LAG3 dimerization. Such mAbs may have different biological effects and therapeutic properties than current mAbs which mainly target the LAG3–MHC class II interaction (Table 1).

While the present emphasis is on generating LAG3 antagonists for cancer immunotherapy that decrease inhibitory signaling, there is growing interest in developing LAG3 agonists to treat autoimmunity by increasing inhibitory signaling (26). The impetus for these efforts comes from genetic deletion and antibody blockade studies of immune checkpoint receptors in mice. Genetic deletion of PD1 or CTLA4 resulted in the development of spontaneous autoimmune symptoms under non-autoimmune prone conditions (75, 76, 77). Although genetic deletion of LAG3 alone did not induce spontaneous autoimmunity, mice with both LAG3 and PD1 deleted experienced more severe multiple organ failure than mice with only PD1 deleted, suggesting that LAG3 and PD1 act synergistically (23, 53). In addition, antibody-mediated depletion of LAG3-positive T cells prevented delayed-type hypersensitivity in non-human primates (78).

Unlike antagonist mAbs, agonist mAbs can be challenging to generate because enhancing receptor activity (*i.e.*, gain-of-function) is generally a more demanding task than inhibiting activity. In fact, only one LAG3-specific agonist mAb (IMP761, a humanized version of the mouse mAb 13E2) has been reported to date (79). No PD1- or CTLA4-specific agonist mAbs have been described. IMP761 exhibited immunosuppressive properties both *in vitro* and *in vivo* in an antigen-specific delayed-type hypersensitivity model in the cynomolgus macaque that mimics psoriasis inflammation, which makes IMP761 a potential candidate for treating T cell-induced autoimmune diseases. Interestingly, IMP761 recognizes an epitope on the LAG3 D1 domain that at least partially overlaps the epitope recognized by the antagonist mAb favezelimab (a humanized version of the mouse mAb 22D2) (32). What makes IMP761 an agonist is unknown, but could involve allosteric effects on LAG3 structure. It is likely that more agonist mAbs with therapeutic potential will be isolated going forward through the application of appropriate screens for detecting enhancement, rather than blockade, of LAG3 signaling activity. If agonist mAbs specific for PD1 or CTLA4 are also discovered, these may be combined with LAG3-specific agonist mAbs, either as separate molecules or as bispecifics, as is being done in cancer therapy (Table 1) (65). In this way, synergistic, or at least additive, immunosuppressive effects may be achieved.

## Conclusion

Recent studies have revealed the dimeric architecture of LAG3 and demonstrated that dimerization is critical for LAG3 inhibitory activity. In addition to MHC class II, its canonical ligand, two new LAG3 ligands have been discovered, FGL1 and the TCR–CD3 complex. However, the relative importance of these ligands is controversial and their hierarchy remains to be defined. In addition, no structural information is yet available for LAG3 bound to any of its biological ligands. Many clinical trials of anti-LAG3 mAbs are underway for treating multiple solid tumors and lymphomas. However, most of these mAbs very likely act by blocking the binding of LAG3 to MHC class II. The recent identification of FGL1 and TCR–CD3 as LAG3 ligands, and the discovery of LAG3 dimerization, open new possibilities to therapeutically target LAG3 using mAbs that block LAG3 interactions with FGL1 or TCR–CD3, or that disrupt LAG3 dimerization. In this way, basic knowledge of LAG3 structure–function relationships may inform the design of more effective strategies to modulate (decrease or increase) LAG3 activity.

## Conflict of interest

The authors declare that they have no conflicts of interest with the contents of this article.

## References

[1] Mariuzza R.A., Agnihotri P., Orban J. (2020). The structural basis of T-cell receptor (TCR) activation: an enduring enigma. J. Biol. Chem..

[2] Francisco L.M., Sage P.T., Sharpe A.H. (2010). The PD-1 pathway in tolerance and autoimmunity. Immunol. Rev..

[3] Romo-Tena J., Gomez-Martin D., Alcocer-Varela J. (2013). CTLA-4 and autoimmunity: new insights into the dual regulator of tolerance. Autoimmun. Rev..

[4] Topalian S.L., Drake C.G., Pardoll D.M. (2015). Immune checkpoint blockade: a common denominator approach to cancer therapy. Cancer Cell.

[5] Hodi F.S., O'Day S.J., McDermott D.F., Weber R.W., Sosman J.A., Haanen J.B. (2010). Improved survival with ipilimumab in patients with metastatic melanoma. N. Engl. J. Med..

[6] Larkin J., Chiarion-Sileni V., Gonzalez R., Grob J.J., Rutkowski P., Lao C.D. (2019). Five-year survival with combined nivolumab and ipilimumab in advanced melanoma. N. Engl. J. Med..

[7] Andrews L.P., Cillo A.R., Karapetyan L., Kirkwood J.M., Workman C.J., Vignali D.A.A. (2022). Molecular pathways and mechanisms of LAG3 in cancer therapy. Clin. Cancer Res..

[8] Chocarro L., Blanco E., Arasanz H., Fernández-Rubio L., Bocanegra A., Echaide M. (2022). Clinical landscape of LAG-3-targeted therapy. Immunooncol. Technol..

[9] Tawbi H.A., Schadendorf D., Lipson E.J., Ascierto P.A., Matamala L., Castillo Gutiérrez E. (2022). Relatlimab and nivolumab versus nivolumab in untreated advanced melanoma. N. Engl. J. Med..

[10] Andrews L.P., Yano H., Vignali D.A.A. (2019). Inhibitory receptors and ligands beyond PD-1, PD-L1 and CTLA-4: breakthroughs or backups. Nat. Immunol..

[11] Triebel F., Jitsukawa S., Baixeras E., Roman-Roman S., Genevee C., Viegas-Pequignot E. (1990). LAG-3, a novel lymphocyte activation gene closely related to CD4. J. Exp. Med..

[12] Ming Q., Celias D.P., Wu C., Cole A.R., Singh S., Mason C. (2022). LAG3 ectodomain structure reveals functional interfaces for ligand and antibody recognition. Nat. Immunol..

[13] Maruhashi T., Okazaki I.M., Sugiura D., Takahashi S., Maeda T.K., Shimizu K. (2018). LAG-3 inhibits the activation of CD4^+^ T cells that recognize stable pMHCII through its conformation-dependent recognition of pMHCII. Nat. Immunol..

[14] Kouo T., Huang L., Pucsek A.B., Cao M., Solt S., Armstrong T. (2015). Galectin-3 shapes antitumor immune responses by suppressing CD8^+^ T cells via LAG-3 and inhibiting expansion of plasmacytoid dendritic cells. Cancer Immunol. Res..

[15] Xu F., Liu J., Liu D., Liu B., Wang M., Hu Z. (2014). LSECtin expressed on melanoma cells promotes tumor progression by inhibiting antitumor T-cell responses. Cancer Res..

[16] Mao X., Ou M.T., Karuppagounder S.S., Kam T.I., Yin X., Xiong Y. (2016). Pathological α-synuclein transmission initiated by binding lymphocyte-activation gene 3. Science.

[17] Wang J., Sanmamed M.F., Datar I., Su T.T., Ji L., Sun J. (2019). Fibrinogen-like protein 1 is a major immune inhibitory ligand of LAG-3. Cell.

[18] Guy C., Mitrea D.M., Chou P.C., Temirov J., Vignali K.M., Liu X. (2022). LAG3 associates with TCR-CD3 complexes and suppresses signaling by driving co-receptor-Lck dissociation. Nat. Immunol..

[19] Zhang Q., Chikina M., Szymczak-Workman A.L., Horne W., Kolls J.K., Vignali K.M. (2017). LAG3 limits regulatory T cell proliferation and function in autoimmune diabetes. Sci. Immunol..

[20] Lucas C.L., Workman C.J., Beyaz S., LoCascio S., Zhao G., Vignali D.A.A. (2011). LAG-3, TGF-β, and cell-intrinsic PD-1 inhibitory pathways contribute to CD8 but not CD4 T-cell tolerance induced by allogeneic BMT with anti-CD40L. Blood.

[21] Goldberg M.V., Drake C.G. (2011). LAG-3 in cancer immunotherapy. Curr. Top. Microbiol. Immunol..

[22] Anderson A.C., Joller N., Kuchroo V.K. (2016). Lag-3, Tim-3, and TIGIT: co-inhibitory receptors with specialized functions in immune regulation. Immunity.

[23] Woo S.R., Turnis M.E., Goldberg M.V., Bankoti J., Selby M., Nirschl C.J. (2012). Immune inhibitory molecules LAG-3 and PD-1 synergistically regulate T-cell function to promote tumoral immune escape. Cancer Res..

[24] Ibrahim R., Saleh K., Chahine C., Khoury R., Khalife N., Cesne A.L. (2023). LAG-3 inhibitors: novel immune checkpoint inhibitors changing the landscape of immunotherapy. Biomedicines.

[25] Aggarwal V., Workman C.J., Vignali D.A.A. (2023). LAG-3 as the third checkpoint inhibitor. Nat. Immunol..

[26] Grebinoski S., Vignali D.A.A. (2020). Inhibitory receptor agonists: the future of autoimmune disease therapeutics?. Curr. Opin. Immunol..

[27] Silberstein J.L., Du J., Chan K.W., Frank J.A., Mathews I.I., Kim Y.B. (2024). Structural insights reveal interplay between LAG-3 homodimerization, ligand binding, and function. Proc. Natl. Acad. Sci. U. S. A..

[28] Mishra A.K., Shahid S., Karade S.S., Agnihotri P., Kolesnikov A., Hasan S.S. (2023). CryoEM structure of a therapeutic antibody (favezelimab) bound to human LAG3 determined using a bivalent Fab as fiducial marker. Structure.

[29] Li Y., Yin Y., Mariuzza R.A. (2013). Structural and biophysical insights into the role of CD4 and CD8 in T cell activation. Front. Immunol..

[30] Li N., Workman C.J., Martin S.M., Vignali D.A. (2004). Biochemical analysis of the regulatory T cell protein lymphocyte activation gene-3 (LAG-3; CD223). J. Immunol..

[31] Huard B., Mastrangeli R., Prigent P., Bruniquel D., Donini S., El-Tayar N. (1997). Characterization of the major histocompatibility complex class II binding site on LAG-3 protein. Proc. Natl. Acad. Sci. U. S. A..

[32] Agnihotri P., Mishra A.K., Agarwal P., Vignali K.M., Workman C.J., Vignali D.A.A. (2022). Epitope mapping of therapeutic antibodies targeting human LAG3. J. Immunol..

[33] MacLachlan B.J., Mason G.H., Greenshields-Watson A., Triebel F., Gallimore A., Cole D.K. (2020). Molecular characterization of HLA class II binding to the LAG-3 T cell co-inhibitory receptor. Eur. J. Immunol..

[34] Baixeras E., Huard B., Miossec C., Jitsukawa S., Martin M., Hercend T. (1992). Characterization of the lymphocyte activation gene 3-encoded protein. A new ligand for human leukocyte antigen class II antigens. J. Exp. Med..

[35] Wang X.X., Li Y., Yin Y., Mo M., Wang Q., Gao W. (2011). Affinity maturation of human CD4 by yeast surface display and crystal structure of a CD4-HLA-DR1 complex. Proc. Natl. Acad. Sci. U. S. A..

[36] Bettini M., Szymczak-Workman A.L., Forbes K., Castellaw A.H., Selby M., Pan X. (2011). Cutting edge: accelerated autoimmune diabetes in the absence of LAG-3. J. Immunol..

[37] Grebinoski S., Zhang Q., Cillo A.R., Manne S., Xiao H., Brunazzi E.A. (2022). Autoreactive CD8^+^ T cells are restrained by an exhaustion-like program that is maintained by LAG3. Nat. Immunol..

[38] Lui Y., Davis S.J. (2018). LAG-3: a very singular immune checkpoint. Nat. Immunol..

[39] Pos W., Sethi D.K., Call M.J., Schulze M.S., Anders A.K., Pyrdol J. (2012). Crystal structure of the HLA-DM-HLA-DR1 complex defines mechanisms for rapid peptide selection. Cell.

[40] Tzeng S.R., Kalodimos C.G. (2009). Dynamic activation of an allosteric regulatory protein. Nature.

[41] Smock R.G., Gierasch L.M. (2009). Sending signals dynamically. Science.

[42] Tzeng S.R., Kalodimos C.G. (2012). Protein activity regulation by conformational entropy. Nature.

[43] Hawse W.F., Gloor B.E., Ayres C.M., Kho K., Nuter E., Baker B.M. (2013). Peptide modulation of class I major histocompatibility complex protein molecular flexibility and the implications for immune recognition. J. Biol. Chem..

[44] van Hateren A., Anderson M., Bailey A., Werner J.M., Skipp P., Elliott T. (2017). Direct evidence for conformational dynamics in major histocompatibility complex class I molecules. J. Biol. Chem..

[45] Ma J., Ayres C.M., Hellman L.M., Devlin J.R., Baker B.M. (2021). Dynamic allostery controls the peptide sensitivity of the Ly49C natural killer receptor. J. Biol. Chem..

[46] Nagdas S.K., Winfrey V.P., Olson G.E. (2016). Two fibrinogen-like proteins, FGL1 and FGL2 are disulfide-linked subunits of oligomers that specifically bind nonviable spermatozoa. Int. J. Biochem. Cell Biol..

[47] Kim M.S., Pinto S.M., Getnet D., Nirujogi R.S., Manda S.S., Chaerkady R. (2014). A draft map of the human proteome. Nature.

[48] Demchev V., Malana G., Vangala D., Stoll J., Desai A., Kang H.W. (2013). Targeted deletion of fibrinogen like protein 1 reveals a novel role in energy substrate utilization. PLoS One.

[49] Li C.Y., Cao C.Z., Xu W.X., Cao M.M., Yang F., Dong L. (2010). Recombinant human hepassocin stimulates proliferation of hepatocytes in vivo and improves survival in rats with fulminant hepatic failure. Gut.

[50] Liu Z., Ukomadu C. (2008). Fibrinogen-like protein 1, a hepatocyte derived protein is an acute phase reactant. Biochem. Biophys. Res. Commun..

[51] Yan J., Ying H., Gu F., He J., Li Y.L., Liu H.M. (2002). Cloning and characterization of a mouse liver-specific gene mfrep-1, up-regulated in liver regeneration. Cell Res..

[52] Maruhashi T., Sugiura D., Okazaki I.M., Shimizu K., Maeda T.K., Ikubo J. (2022). Binding of LAG-3 to stable peptide-MHC class II limits T cell function and suppresses autoimmunity and anti-cancer immunity. Immunity.

[53] Okazaki T., Okazaki I.M., Wang J., Sugiura D., Nakaki F., Yoshida T. (2011). PD-1 and LAG-3 inhibitory co-receptors act synergistically to prevent autoimmunity in mice. J. Exp. Med..

[54] Shrive A.K., Moeller J.B., Burns I., Paterson J.M., Shaw A.J., Schlosser A. (2014). Crystal structure of the tetrameric fibrinogen-like recognition domain of fibrinogen C domain containing 1 (FIBCD1) protein. J. Biol. Chem..

[55] Leppänen V.M., Saharinen P., Alitalo K. (2017). Structural basis of Tie2 activation and Tie2/Tie1 heterodimerization. Proc. Natl. Acad. Sci. U. S. A..

[56] Held W., Mariuzza R.A. (2008). *Cis* interactions of immunoreceptors with MHC and non-MHC ligands. Nat. Rev. Immunol..

[57] Held W., Mariuzza R.A. (2011). *Cis–trans* interactions of cell surface receptors: biological roles and structural basis. Cell. Mol. Life Sci..

[58] Razi N., Varki A. (1998). Masking and unmasking of the sialic acid-binding lectin activity of CD22 (Siglec-2) on B lymphocytes. Proc. Natl. Acad. Sci. U. S. A..

[59] Collins B.E., Blixt O., DeSieno A.R., Bovin N., Marth J.D., Paulson J.C. (2004). Masking of CD22 by *cis* ligands does not prevent redistribution of CD22 to sites of cell contact. Proc. Natl. Acad. Sci. U. S. A..

[60] Doucey M.A., Scarpellino L., Zimmer J., Guillaume P., Luescher I.F., Bron C. (2004). *Cis* association of Ly49A with MHC class I restricts natural killer cell inhibition. Nat. Immunol..

[61] Masuda A., Nakamura A., Maeda T., Sakamoto Y., Takai T. (2007). *Cis* binding between inhibitory receptors and MHC class I can regulate mast cell activation. J. Exp. Med..

[62] Kim P.W., Sun Z.Y., Blacklow S.C., Wagner G., Eck M.J. (2003). A zinc clasp structure tethers Lck to T cell coreceptors CD4 and CD8. Science.

[63] Feller B., Fallon A., Luo W., Nguyen P.T., Shlaifer I., Lee A.K. (2023). α-Synuclein preformed fibrils bind to β-neurexins and impair β-neurexin-mediated presynaptic organization. Cells.

[64] Emmenegger M., De Cecco E., Hruska-Plochan M., Eninger T., Schneider M.M., Barth M. (2021). LAG3 is not expressed in human and murine neurons and does not modulate a-synucleinopathies. EMBO Mol. Med..

[65] Luke J.J., Patel M.R., Blumenschein G.R., Hamilton E., Chmielowski B., Ulahannan S.V. (2023). The PD-1- and LAG-3-targeting bispecific molecule tebotelimab in solid tumors and hematologic cancers: a phase 1 trial. Nat. Med..

[66] Gutierrez A.A., Grosso J., Hill C.M., Selby M., Lewis K.E., inventors; Bristol-Myers Squibb Co., assignee (August 6, 2015).

[67] Liang L., Fayadat-Dilman L., De Waal Malefyt R., Raghunathan G., inventors; Merck Sharp & Dohme Corp, assignee (February 25, 2016).

[68] Zettl M., Lorenz I., Schaaf O., Wurm M., Fortin J.-F., Brodeur S., inventors; Boehringer Ingelheim International GmbH, assignee (November 23, 2017).

[69] Triebel F., Brignone C., Blattler W.A., Mataraza J.M., Sabatos-Peyton C.A., Chang H.W., inventors; Novartis Ag, Immutep S.A., assignees (September 17, 2015).

[70] Triebel F., Brignone C., inventors; Immutep S.A., assignee (March 9, 2017).

[71] Bhagwat B., Cherwinski H., Sathe M., Seghezzi W., McClanahan T.K., de Waal Malefyt R. (2018). Establishment of engineered cell-based assays mediating LAG3 and PD1 immune suppression enables potency measurement of blocking antibodies and assessment of signal transduction. J. Immunol. Methods.

[72] Ascione A., Arenaccio C., Mallano A., Flego M., Gellini M., Andreotti M. (2019). Development of a novel human phage display-derived anti-LAG3 scFv antibody targeting CD8^+^ T lymphocyte exhaustion. BMC Biotechnol..

[73] Workman C.J., Rice D.S., Dugger K.J., Kurschner C., Vignali D.A. (2002). Phenotypic analysis of the murine CD4-related glycoprotein, CD223 (LAG-3). Eur. J. Immunol..

[74] Maeda T.K., Sugiura D., Okazaki I.M., Maruhashi T., Okazaki T. (2019). Atypical motifs in the cytoplasmic region of the inhibitory immune co-receptor LAG-3 inhibit T cell activation. J. Biol. Chem..

[75] Nishimura H., Nose M., Hiai H., Minato N., Honjo T. (1999). Development of lupus-like autoimmune diseases by disruption of the PD-1 gene encoding an ITIM motif-carrying immunoreceptor. Immunity.

[76] Tivol E.A., Borriello F., Schweitzer A.N., Lynch W.P., Bluestone J.A., Sharpe A.H. (1995). Loss of CTLA-4 leads to massive lymphoproliferation and fatal multiorgan tissue destruction, revealing a critical negative regulatory role of CTLA-4. Immunity.

[77] Waterhouse P., Penninger J.M., Timms E., Wakeham A., Shahinian A., Lee K.P. (1995). Lymphoproliferative disorders with early lethality in mice deficient in Ctla-4. Science.

[78] Poirier N., Haudebourg T., Brignone C., Dilek N., Hervouet J., Minault D. (2011). Antibody-mediated depletion of lymphocyte-activation gene-3 (LAG-3(+))-activated T lymphocytes prevents delayed-type hypersensitivity in non-human primates. Clin. Exp. Immunol..

[79] Angin M., Brignone C., Triebel F. (2020). A LAG-3-specific agonist antibody for the treatment of T cell-induced autoimmune diseases. J. Immunol..

[80] Albrecht L.J., Livingstone E., Zimmer L., Schadendorf D. (2023). The latest option: nivolumab and relatlimab in advanced melanoma. Curr. Oncol. Rep..

[81] Zettl M., Wurm M., Schaaf O., Mostböck S., Tirapu I., Apfler I. (2022). Combination of two novel blocking antibodies, anti-PD-1 antibody ezabenlimab (BI 754091) and anti-LAG-3 antibody BI 754111, leads to increased immune cell responses. Oncoimmunology.

[82] Jiang H., Ni H., Zhang P., Guo X., Wu M., Shen H. (2021). PD-L1/LAG-3 bispecific antibody enhances tumor-specific immunity. Oncoimmunology.

[83] Edwards C.J., Sette A., Cox C., Di Fiore B., Wyre C., Sydoruk D. (2022). The multi-specific V_H_-based Humabody CB213 co-targets PD1 and LAG3 on T cells to promote anti-tumour activity. Br. J. Cancer.

[84] Sung E., Ko M., Won J.Y., Jo Y., Park E., Kim H. (2022). LAG-3xPD-L1 bispecific antibody potentiates antitumor responses of T cells through dendritic cell activation. Mol. Ther..

